# Unpacking willpower in unassisted smoking cessation: a qualitative analysis reveals a systematic profile of situational and cognitive strategies

**DOI:** 10.1080/21642850.2026.2644658

**Published:** 2026-03-14

**Authors:** Effie Marathia, Sheila Duffy, Abigail Stephen, Kimberly R. More, Blair Saunders

**Affiliations:** a Division of Psychology, School of Humanities, Social Sciences, and Law, University of Dundee, Dundee, United Kingdom; b Action on Smoking and Health (ASH) Scotland, Edinburgh, United Kingdom; c The Rowett Institute, School of Medicine, Medical Sciences and Nutrition, University of Aberdeen, Aberdeen, United Kingdom; d School of Psychology and Neuroscience, University of St Andrews, St Andrews, United Kingdom

**Keywords:** Unassisted smoking cessation, self-regulation strategies, behavior change techniques, public health policy

## Abstract

**Background:**

Over half of those who quit smoking do so without formal assistance, yet the psychological processes supporting unassisted cessation remain little understood. Success is often attributed to willpower, an umbrella term that lacks explanatory precision and obscures the underlying tractable processes. Drawing on the Process Model of Self-Regulation and the Behavior Change Technique (BCT) Taxonomy, this study aimed to identify the concrete strategies that enable individuals to quit smoking unassisted, thereby clarifying what willpower might look like in practice.

**Materials and methods:**

Thirty-two participants who had successfully quit smoking without formal support participated in semi-structured interviews. Inductive content analysis identified key challenges, while deductive coding mapped strategies addressing these challenges to the Process Model of Self-Regulation and the BCT Taxonomy.

**Results:**

Participants’ accounts reflected a diverse range of strategies, averaging seven distinct BCTs, spanning the Situation Selection and Modification, Attention Redeployment, and Cognitive Change stages from the Process Model. Common BCTs included avoiding environmental triggers, substituting smoking with alternative behaviors, and seeking social support. In contrast, Response Modulation (e.g. ‘just say no’) accounted for only 1% of the data.

**Conclusion:**

Unassisted quitters drew from a sophisticated repertoire of strategies that are actionable, teachable, and embedded within the individual’s physical and social environment. The qualitative methodology used in this study offers an understanding of the lived experiences of self-quitters, potentially informing public health interventions that integrate individual and system-level approaches to behavior change that extend beyond brute-force willpower.

## Introduction

Tobacco poses a major threat to public health, causing over 7 million deaths and costing $1.4 trillion annually as estimated in 2012 (Goodchild et al., 2018; World Health Organisation, 2025). The World Health Organisation’s (WHO) *Global Action Plan for the Prevention and Control of Non-Communicable Diseases* aims to reduce smoking prevalence by 30% by the end of 2025 relative to 2010 levels; however, this target is projected to be missed by approximately three years (WHO, 2019, 2025). While these statistics motivate formal cessation interventions, over half of those who quit do so without formal assistance (~45–70% across studies and countries; Edwards et al., 2014; Smith et al., 2015b; Smoking Toolkit Study, 2024b; 2025). The desire to demonstrate commitment and resoluteness, as well as to avoid the medicalization of cessation, might explain *why* individuals prefer unassisted quitting (Smith et al., 2015a, 2015b). However, the processes through which individuals achieve unassisted cessation remain poorly understood. We conducted qualitative interviews to explore the strategies used by unassisted quitters, asking whether such quitting is associated with a systematic profile of techniques. Understanding these processes, we believe, could provide insights into what drives successful cessation without formal support, potentially informing public health strategies to enhance quitting rates.

*Willpower* is frequently evoked to explain unassisted cessation (Smith et al., 2015a, 2015b; Stewart, 1999). A systematic review of qualitative studies of unassisted cessation (Smith et al., 2015a), found that willpower was often invoked as a defining ingredient of success but was rarely unpacked in terms of underlying processes. As such, willpower alone lacks explanatory power to truly understand unassisted cessation for several reasons. First, willpower has been defined inconsistently (Ludwig, 1985; Smith et al., 2015a; Stewart, 1999), making findings across studies difficult to compare. Second, accounts that attribute successful unassisted cessation to willpower often lack specificity: individuals are suggested to use willpower to quit, while their willpower is diagnosed by the fact they have already quit (cf., Stewart, 1999), which might obscure the psychological or behavioural processes that enabled success. Therefore, a key motive underlying this research was to apply contemporary self-regulation and behavior change frameworks to unpack the processes that may underlie lay accounts of willpower in unassisted cessation (Duckworth et al., 2016; Duckworth et al., 2014; Michie et al., 2013).

Self-regulation refers to a range of psychological processes that facilitate self-directed goal pursuit, either by encouraging goal-consistent actions or overcoming temptations (Carver & Scheier, 1998; Inzlicht et al., 2021). This makes self-regulation an ideal lens for studying unassisted cessation, as this method of quitting is self-deployed in relation to the cessation goal. Without reference to smoking, self-regulation theorists have independently challenged the centrality of willpower in self-control (Fujita et al., 2020). In self-regulation, willpower has often been synonymous with inhibition and self-control, referring to the ‘brute-force’ ability to resist temptation (Baumeister, 1998; Mischel, 1996; Muraven & Baumeister, 2000). However, many instances attributed to successful willpower (e.g. ‘I resisted the urge to smoke’) might identify an outcome—abstinence despite temptation—that could be achieved through a wide array of processes (e.g. distraction or reminding yourself why you want to quit; Werner et al., 2022). Thus, it is important to move beyond the single category of willpower to consider a wider toolbox of strategies that might underlie health promotion (Fujita et al., 2020).

To explore the self-regulatory toolbox associated with unassisted cessation, we drew from the Process Model of Self-Regulation (Duckworth et al., 2016; Duckworth et al., 2014), which categorizes strategies based on their focus on external situations (the physical or social environment) or internal cognitions (attention, thoughts, and feelings). These can be further delineated into strategy categories that intervene at different stages of desire generation. Situational strategies include *situation selection,* where individuals place themselves in environments conducive to their goals, and *situation modification,* which involves altering one’s environment to support goal progress (Inzlicht et al., 2021). Attention Redeployment strategies involve shifting attention away from temptations, for example, by distraction (Milyavskaya et al., 2021), while Cognitive Change strategies alter the meaning of the desire (Webb et al., 2012). Finally, Response Modulation strategies are a late intervention that might stop a behavior already initiated, for example, stopping your hand from reaching for a cigarette (Gross, 2015). The Process Model predicts that early situational interventions are more effective, as they prevent exposure to temptation (Duckworth et al., 2016). Given the strong environmental and social triggers associated with smoking (Conklin et al., 2010; Conklin et al., 2013; Conklin et al., 2008; Stevenson et al., 2017), situational strategies may be particularly prevalent among those who quit unassisted.

While self-regulation strategies offer insights into unassisted cessation, they also reflect higher-level categories within which many specific tactics are nested (Werner & Ford, 2023). Situation modification, for example, could be realized by hiding cigarettes or positioning yourself away from smokers at social events. These lower-level, concrete processes are identified as tactics in the broader self-regulation literature (Werner & Ford, 2023; Werner et al., 2022). The range of lower-level tactics individuals may use to regulate their behavior is potentially infinite, yet a formal taxonomy mapping these is currently lacking in self-regulation research (Ladis et al., 2023; Werner et al., 2022). To this end, we aligned terminology with the Behavior Change Technique (BCT) Taxonomy (v1) (Michie et al., 2013) to formalize and operationalize these lower-level tactics. BCTs are defined as ‘observable, replicable, and irreducible components of an intervention designed to regulate behavior’ or as the ‘active ingredients’ of behavior change (Michie et al., 2013, p. 82).

BCTs have been instrumental in evaluating structured interventions delivered across diverse settings, such as post-discharge from smoke-free institutions including hospitals, prisons, and mental health clinics (Shoesmith et al., 2021), as well as in pre-surgical cessation programs (Prestwich et al., 2017). These studies identified techniques such as goal setting, progress monitoring, and social support as effective in facilitating cessation. However, these investigations typically focus on assisted quitting—i.e. contexts where individuals benefit from structured professional or institutional input. In contrast, we know relatively little about what unassisted quitters actually do when quitting without formal support. Understanding this lived process may reveal how people self-structure their cessation attempts, and whether these align with or diverge from the strategies identified in intervention-based research. Accordingly, we explored a subset of BCTs that can be self-enacted without a formalized intervention (Knittle & Fidrich, 2023; Hankonen, 2021; Knittle et al., 2020). For example, an unassisted quitter might enact situational strategies though BCTs such as avoiding shops that sell cigarettes (12.3 Avoiding/reducing exposure to cues) or locking cigarettes away (7.3 Remove access to the reward).

Using interview data, we document the in-the-moment strategies and tactics people use when quitting unassisted, offering insights that cannot be captured through survey tallies alone (e.g. Milyavskaya et al., 2021). By asking participants to narrate their quitting journey, our qualitative approach provides a naturalistic, bottom-up account of the challenges they faced and how they responded to them. This allowed us to identify the broad themes characterizing unassisted cessation while also systematizing the concrete techniques participants used to navigate difficulties. Because identifying themes inevitably compresses detail, we complemented this inductive analysis with a deductive mapping of participants’ strategies onto established self-regulation and behavior change frameworks. Linking these descriptions to specific BCTs and Process Model strategies clarifies the lower-level processes that may underpin behavior change, helping to unpack willpower in unassisted cessation. Using this existing taxonomy also strengthens bridges between social-personality theories of self-regulation and applications within health psychology, offering a more precise and theoretically grounded account of how unassisted quitting might be achieved.

## Materials and methods

### Overview

**Figure 1.**
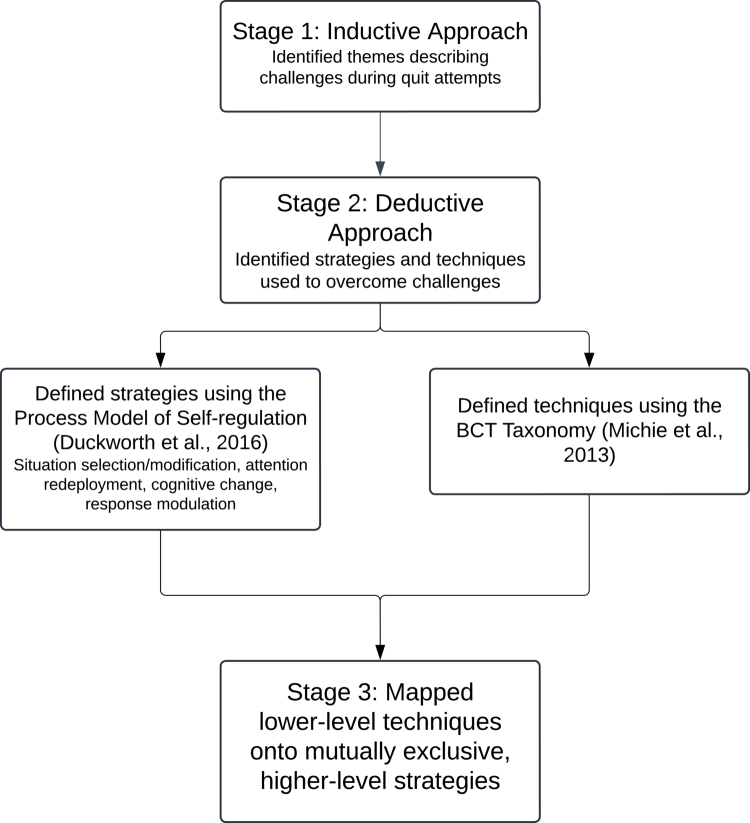
Summary of the analytic process.

This qualitative interview study explored and mapped strategies involved in unassisted cessation (Figure 1). The study received ethical approval from the University of Dundee School of Social Sciences and Humanities Research Ethics Committee (UOD-SOSS-PSY-PG-2021-104) and was pre-registered on OSF (Appendix 1).

Prior to interviews, co-design involved consulting with the Tayside Medical Science Centre Patient and Public Involvement (PPI) group and our collaborating organization, health charity Action on Smoking and Health (ASH) Scotland, to refine the interview questions. The PPI group included four individuals: two current smokers and two former smokers who quit unassisted (see Appendix 2). Three individuals from ASH Scotland (one co-author and two team members) contributed to developing the interview schedule through conceptual discussions, feedback on schedule drafts, and practice interviews. Their contribution was advisory; personal lived experience information was not collected. ASH Scotland is a charity working with the Scottish Government, the NHS, and local communities to tackle the harms caused by tobacco.

In-depth semi-structured interviews were conducted via Zoom between August 2021 and July 2022, averaging 58.8 minutes (range: 31-92 minutes). Participants discussed multiple topics related to quitting; however, for the current analysis, we specifically focused on questions around strategy use (see full interview schedule in Appendix 3).

### Participants

Participants were recruited through ASH Scotland’s network, including third-sector advocacy, community organizations, local government, and online platforms such as the Call for Participants website (*Call for Participants,* 2024). Recruitment combined online advertisements (via social media, mailing lists, and discussion forums) with physical posters displayed in community centres across Dundee, with particular efforts to reach communities from lower socioeconomic backgrounds. As such, although participation was self-selecting, recruitment was purposive in intent, aiming to capture a diverse range of unassisted quitters in terms of socioeconomic background. Example recruitment materials are provided on the study’s OSF page.

Inclusion criteria included being over 18 years old, having quit unassisted 0.5 to 10 years prior to data collection, and being residing in Scotland. Participants were reimbursed with a £10 supermarket voucher. Written consent was provided prior to the interview and reaffirmed at its start.

For recruitment purposes, *unassisted cessation* was defined as having quit smoking ‘on your own, without help from a health professional or medication’, as stated in the recruitment materials. This definition excluded the use of formal cessation aids such as nicotine replacement therapy, prescribed pharmacological support, or behavioral counselling at the time of quitting. No exclusions were made based on current use of other tobacco products, e-cigarettes, or substances such as alcohol, as these were beyond the scope of the study’s focus on unassisted cigarette cessation.

Smoking status was verified via self-report, consistent with established guidance indicating that self-report is generally accurate and appropriate in many cessation research contexts, particularly when biochemical verification is not feasible or required for the study aims (Patrick et al., 1994; SRNT Subcommittee on Biochemical Verification, 2002). Each participant completed brief smoking-related questions prior to the interview, and responses were cross-checked with information provided during the interview (e.g. when they quit, smoking duration). Several procedures were used to ensure data integrity and minimise fraudulent participation, including: conversational screening of location and background at the start of the interview, consistency checks between survey and interview responses, encouraging participants to keep cameras on, and assessing depth of engagement particularly using open questions. These approaches, informed by guidance for online qualitative research (Jenner & Myers, 2019; Simone, 2019), balanced appropriate safeguards with participant comfort and ethical feasibility.

Thirty-four unassisted quitters expressed interest; however, two did not respond to further correspondence, resulting in 32 interviews (20 male, 12 female, average age 37 years, range 20−74). Fourteen participants identified as White Scottish, with others identifying as Black/Black British (*n *= 12), English (*n *= 2), Mixed (*n *= 2), Asian/Asian British (*n *= 1), and European (*n *= 1) (Table 1). Although we preregistered analyses incorporating socioeconomic status, most participants (*n *= 27) identified within the middle range, limiting meaningful comparisons.

**Table 1.** Participant characteristics (N = 32).

Characteristic	N	Percentage (%)
**Gender**		
Female	12	37.5
Male	20	62.5
**Age**		
Range	20–74	
Mean (SD)	37.2 (14.6)	
**Ethnic Group**		
White Scottish	14	43.8
Black/Black British	12	37.5
English	2	6.3
Mixed	2	6.3
Asian/Asian British	1	3.1
European	1	3.1
**Occupation status**		
Working full-time	11	34.4
Working part-time	9	28.1
Full-time student	4	12.5
Unemployed	5	15.6
Retired	2	6.3
On sickness	1	3.1
**Highest level of education**		
Primary school	1	3.1
Secondary school up to 16 years	2	6.3
Secondary school beyond 16 years	6	18.8
Further education	5	15.6
University undergraduate degree	14	43.8
Taught postgraduate degree	4	12.5
**Subjective socioeconomic status**		
Bottom of the ladder (1−3)	2	6.3
Middle of the ladder (4−7)	27	84.4
Top of the ladder (8−10)	2	6.3
Prefer not to say	1	3.1
Mean (SD)	5.6 (1.5)	
**Years as a smoker**		
Range	2–53	
Mean (SD)	14.6 (10.8)	
**Number of cigarettes smoked/day**		
Range	4-50	
Mean (SD)	15.0 (10.8)	
**Time since quit (months)**		
Range	6–120	
Mean (SD)	38.4 (35.9)	

### Interview schedule

The semi-structured interview schedule was organized around the stages of the Process Model of Self-Regulation (Duckworth et al., 2016; Duckworth et al., 2014), ensuring balanced coverage of situational and cognitive strategies, as well as response modulation (Appendix 3). To achieve this balance, an equivalent number of questions was included for each strategy category (e.g. three questions on situational strategies and three on cognitive strategies). Across categories, participants were asked parallel questions to encourage reflection on different forms of self-regulation (e.g. modifying surroundings, seeking support, distraction, reframing thoughts). For instance, situational strategies were explored through prompts such as, ‘Were there any ways in which you changed your surroundings, or deliberately put yourself in situations, so that you could avoid things or certain people that reminded you of smoking? […] Can you tell me why you did that?’, while cognitive strategies were explored through questions like, ‘Was there any way that you changed how you thought about smoking during quitting? […] Can you tell me why you did that?’.

Response modulation was addressed through the following question: ‘Did you ever just say no when offered a cigarette? How was that for you?’. Participants’ responses to this question (e.g. ‘I said no’; ‘I wasn’t going to ruin it’; ‘Just said to myself ‘don’t smoke’’) confirmed that while inhibitory control was part of their quitting experience, they typically situated it within a broader set of situational and cognitive strategies. The interview design therefore blended deductive elements (to ensure theoretical coverage) with inductive openness, to allow participants to elaborate freely on their experiences in their own terms.

### Data analysis

Our analysis followed a two-stage approach, combining inductive and deductive content analysis as described by Elo and Kyngas (2008). The inductive stage captured how unassisted quitters themselves conceptualized their experiences—i.e. the broad problems they confronted and the ways they described managing the process—in their own words. Participants described quitting in rich and varied ways, using metaphors such as ‘mind disease’ or talking about sensations in their lungs, future peace of mind, or the need to ‘keep busy’. The subsequent deductive stage then mapped how those problems were resolved, by aligning participants’ accounts with established self-regulation and behavior change frameworks. In doing so, the analysis reinterpreted participants’ accounts through a complementary expert vocabulary and made visible the psychological and behavioral ‘building blocks’ that may underlie willpower during unassisted cessation. This design therefore prioritized conceptual specificity (i.e. the psychological and behavioral processes underlying quitting) over a fully inductive narrative analysis. For clarity, we define key terms below:

A **theme** is a broad, overarching category capturing significant patterns or topics within the data, representing major challenges and events during cessation (e.g. ending relationships with friends who smoked). Themes are mutually exclusive and refer to broad-level events within the person’s quitting journey.

A **strategy** is a higher-level, goal-directed class of methods—based on a finite set of pre-defined strategies—that participants used to manage cessation challenges, corresponding to stages defined by the Process Model of Self-regulation.

A **technique** is an ‘active ingredient’ of behavior change identified in the BCT Taxonomy, representing the lowest-level actionable steps taken during cessation. These techniques were nested within strategies and equivalent to what other authors have identified as ‘tactics’ (e.g. Werner & Ford, 2023).

We preregistered sharing transcripts with participants for accuracy cheques. However, due to transcript length (15−30 pages) and non-responses from requests from initial participants, this approach was dropped. We incorporated direct quotes from participants throughout our analysis. Quotes were selected to illustrate key themes and convey the range of common and unique perspectives within each theme.

#### Validity and trustworthiness

Throughout the analytical process, two authors independently reviewed and coded early transcripts, compared interpretations, and refined the coding framework through discussion and clarifying differences. A third author subsequently reviewed the coding framework and applied it to a subset of transcripts to ensure that it could be used consistently by others.

Emerging inductive themes and corresponding quotations were discussed extensively within the research team and presented to health psychologists, smoking cessation advisors, policymakers and public health academics. These consultations helped refine theme boundaries, test the resonance of interpretations, and confirm the relevance of findings for applied practice and public health policy. Although participants did not provide feedback on findings, this iterative team-based analysis and consultation with domain experts and practitioners offered a robust form of external validation and reflexive scrutiny.

Because the study’s primary aim was to explore the correspondence between lay accounts of unassisted quitting and expert frameworks of self-regulation and behavior change, rather than to develop grounded theory, the emphasis was placed on analytic transparency, collaborative review, and conceptual alignment rather than on theoretical sampling or saturation.

#### Inductive content analysis to identify themes

First, the lead author familiarized themselves with the transcripts and conducted open coding, resulting in 267 codes. These codes were organized into ten mutually exclusive themes using NVivo R14.23.0. To ensure reliability, a co-author independently sorted 40 randomly selected interview extracts (15% of codes) into themes (Cohen’s *k* = .64—substantial agreement). Discrepancies were resolved through discussion between the two coders, and a third author was consulted in cases requiring further resolution. Consequently, the original ten themes were refined into seven, verified through a subsequent independent coding exercise (*k = *.65).

#### Deductive content analysis

The first author conducted the initial coding, reviewed by a co-author, resulting in 84% agreement on the BCT coding. For most of the remaining 16%, the co-author suggested that data coded as *behavior substitution* should also be coded as *habit reversal,* as the behaviors described to substitute smoking occurred regularly. When integrating the Process Model and BCT taxonomy, the lead author mapped each of the 33 BCTs identified to one of the four Process Model strategies (Situational Strategies including selection and modification, Attention Redeployment, Cognitive Change, and Response Modulation). Definitions of BCTs identified in our data can be found in Appendix 4.

An additional co-author reviewed this mapping, with 82% agreement. Divergences primarily involved reassigning six BCTs (*graded tasks*, *problem-solving*, *goal setting*—*behavior*, *goal setting*—*outcome*, *action planning*, and *self-monitoring*) from Situational Strategies to Cognitive Change (see also Hennecke et al., 2019). However, transcripts showed that these cognitive strategies were often tightly connected to external stimuli or objects (e.g. using an app for self-monitoring; Duckworth et al., 2018; Hennecke et al., 2019).

#### Researcher positionality and reflexivity

All interviews were conducted by the first author, a White woman with a background in Health Psychology who was in her mid-20s at the time of data collection. Although she has never been a regular smoker, she grew up in a country where smoking is highly prevalent and in a household where indoor smoking was common. These experiences informed her understanding of the social and cultural contexts surrounding smoking and shaped the perspectives she brought to both data collection and analysis.

The analytical team comprised researchers with disciplinary training in Health and Social and Personality Psychology with mixed personal experiences of smoking. Throughout the analysis, the team engaged in regular discussions to reflect on emerging interpretations, identify potential assumptions arising from disciplinary or personal backgrounds, and consider how their perspectives might shape the coding. Independent coding, comparison of interpretations, and iterative refinement of the coding framework supported reflexive awareness and helped mitigate individual biases, enhancing the credibility and trustworthiness of the findings.

## Results

### Overview of the analyses

The inductive analysis explored how participants conceptualized the challenges of quitting smoking unassisted and the ways they navigated these challenges (Table 2). We generated 267 codes containing 442 references, whereby a single code could include multiple references from different participants (e.g. code ‘Gradually reducing nicotine’ included five references). Percentages were calculated using the number of references as the unit of analysis. NVivo pools references contributing to each code and theme, providing an indication of how frequently participants discussed particular strategies. This approach reflects the quantity of narratives underlying each theme.

**Table 2.** Inductive content analysis themes.

Theme	Participants (*N *= 32)	References (*N*)	Frequency in data (%)	Description	Example quote
Doing things differently	27	134	30.3	Changing habits and filling time with activities when quitting smoking.	‘I changed my route to work […]. […] if I always used to turn right, it’s a seven-minute walk to work, just enough time to have a cigarette. Whereas I was like, I'll try something different, so I went the other way.’
Avoiding tempting situations	24	74	16.7	Staying away from smoking friends, social events, and familiar places associated with smoking.	‘[…] I previously had lunch in the canteen, [but] I would make a packed lunch, and I would go and sit somewhere else […]. And that kind of saves me saying to people leave me alone […].’
Seeking help from friends and family	22	51	11.5	Relying on support from social networks to overcome smoking urges.	‘[My family and friends] encourage[d] me to carry on […]. They were there all along and I’m very grateful for having such friends and family because I really needed to know that they’re supportive of what I’m doing.’
Gradual ways of quitting smoking	24	45	10.2	Gradually reducing cigarette intake, saving cigarette money, and goal-setting.	‘[…] I just put that cigarette money into a savings account. So I've been doing that for three years. I just put £20 away a month […]. And it still is a massive thing, because you see that money piling up.’
Saying no to cigarettes	25	45	10.2	Facing peer pressure, participants refused offers and reinforced decisions with both verbal refusals and mental affirmations.	‘I kept saying to myself: ‘no cigarettes, no cigarettes’ […]. […] is that cigarette really worth lying on my back with them oxygen tanks? No […].’
Changing thoughts around smoking	22	41	9.3	Using negative visualizations, future-oriented thinking, self-encouragement, and flexible quitting approaches.	‘When I was feeling that I have to smoke […], I started thinking about that feeling when I'm walking uphill after smoking a cigarette, which I felt was horrible. So thinking about those things kept me off from smoking.’
Setting boundaries, ending friendships, and establishing new ones	15	23	5.2	Reducing or ending relationships with smoker friends and integrating into new, healthier social groups.	‘[…] I found it too much to socialise with [heavy smokers] because the pressure was too much for me to go down that route. So I don't see them so much as I would have done before, had I kept on with the smoking.’

Note: Percentages represent the proportion of references assigned to each theme (*N *= 442 total references). Each reference was counted once and assigned to only one theme (themes were mutually exclusive).

For the content analysis, percentages were calculated out of the total references across all themes (*N *= 442). Themes were mutually exclusive, so each reference contributed to only one theme. However, applying the Process Model and BCT frameworks increased the total number of coded references, as a single reference could map to multiple strategies or BCTs. Accordingly, percentages for the Process Model and BCT frameworks were calculated relative to the total number of references at the framework level.

The most prominent theme ‘Doing things differently’ (134/442, 30.3%) illustrated how participants reshaped their daily routines. ‘Avoiding tempting situations’ (74/442, 16.7%) captured efforts to steer clear of environments encouraging smoking. Participants sought support from friends and family, employing strategies ranging from emotional encouragement to practical assistance (51/442, 11.5%). *‘*Gradual ways of quitting smoking*’* and *‘*Saying no to cigarettes*’* (each 45/442, 10.2%) highlighted methods for reducing nicotine intake or resisting temptation. Some focused on *‘*Changing thoughts around smoking*’* (41/442, 9.3%), reframing their mindset to emphasize the benefits of quitting, while others set firm boundaries, even ending relationships hindering their progress (23/442, 5.2%).

**Figure 2.**
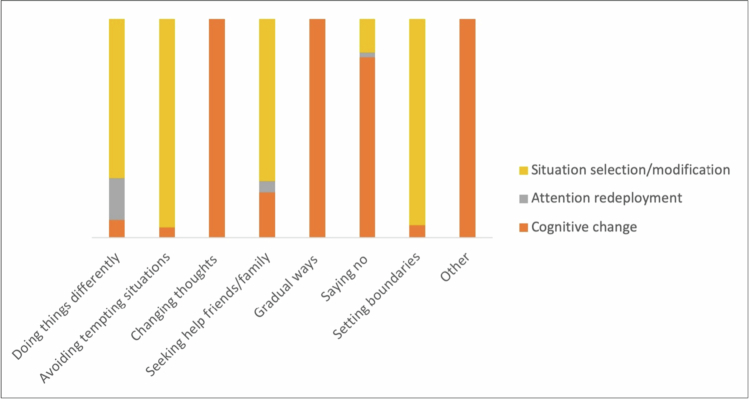
Distribution of process model strategies across themes.

**Figure 3.**
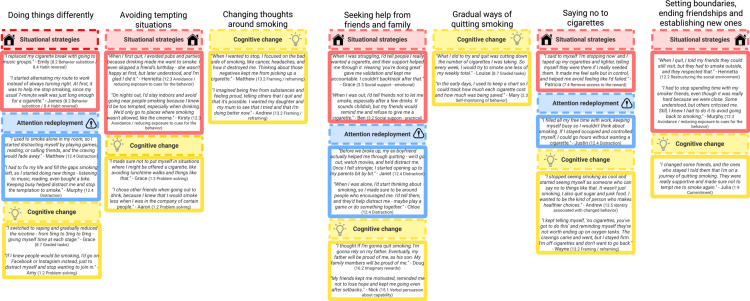
Examples of process model strategies and BCTs identified within themes.

The deductive analysis mapped participants’ strategies onto the Process Model of Self-Regulation, accounting for 93.4% of our data (Figure 2), and the BCT Taxonomy, which accounted for 94.1%, offering a structured understanding of their techniques (Table 3, Figure 3). Table 3 summarises the mapping of Process Model strategies and associated BCTs across themes. While higher-level categories such as Situational Strategies appeared under several themes, the specific BCTs and contexts in which they were enacted differed. For instance, Situational Strategies in ‘Doing things differently’ reflected behavioral substitutions (e.g. replacing cigarettes with other activities), whereas in ‘Avoiding tempting situations’ they centred on avoiding smoking cues and restructuring social environments.

**Table 3.** Summary of strategies, BCTs, and frequencies identified in the deductive content analysis.

Theme	Process Model Category	Frequency in data (%)	BCTs	Participants/Frequency in data (%)
Doing things differently	Situational Strategies	71.5	Behavior substitution	27/34.8
			Habit reversal	27/34.8
			Avoiding/reducing exposure to cues	6/1.9
	Attention Redeployment	19.0	Distraction	21/19.0
	Cognitive Change	7.9	Graded tasks	6/4.9
			Problem-solving	8/3.0
			Reducing negative emotions (not assigned to a stage)	3/1.6
Avoiding tempting situations	Situational Strategies	94.8	Avoiding/reducing exposure to cues	24/70.1
			Restructuring the social environment	12/15.5
			Restructuring the physical environment	6/7.2
			Behavior substitution	1/1.0
			Habit reversal	1/1.0
	Cognitive Change	4.6	Problem-solving	4/4.6
Seeking help from friends and family	Situational Strategies	74.0	Social support (emotional)	18/53.4
			Social support (unspecified)	6/12.3
			Social support (practical)	3/8.2
	Cognitive Change	20.5	Verbal persuasion about capability	5/11.0
			Imaginary reward	2/4.1
			Framing/reframing	2/2.7
			Commitment	1/1.4
			Imaginary punishment	1/1.4
	Attention Redeployment	5.5	Distraction	3/5.5
Gradual ways of quitting smoking	Cognitive Change	100	Graded tasks	21/49.3
			Self-monitoring of behavior	7/14.9
			Goal setting (behavior)	4/10.4
			Self-reward	4/7.5
			Goal setting (outcome)	2/4.5
			Action planning	2/4.5
			Material reward (behavior)	2/4.5
			Instruction on how to perform the behavior	1/3.0
			Non-specific reward	1/1.5
Saying no to cigarettes	Cognitive Change	82.6	Identity associated with changed behavior	19/60.9
			Framing/reframing	1/8.7
			Imaginary punishment	1/8.7
			Self-talk	2/4.3
	Situational Strategies	15.2	Behavior substitution	2/6.5
			Habit reversal	2/6.5
			Remove access to reward	1/2.2
	Attention Redeployment	2.2	Distraction	1/2.2
Setting boundaries, ending friendships, and establishing new ones	Situational Strategies	94.4	Restructuring the social environment	15/63.9
			Avoiding/reducing exposure to cues	9/30.6
	Cognitive Change	5.6	Goal setting (behavior)	1/2.8
			Commitment	1/2.8
Changing thoughts around smoking	Cognitive Change	100	Framing/reframing	22/49.4
			Imaginary punishment	7/18.2
			Imaginary reward	7/9.1
			Salience of consequences	4/6.5
			Self-talk	4/5.2
			Problem-solving	2/2.6
			Goal setting (outcome)	2/2.6
			Information about health consequences	2/2.6
			Identity associated with changed behavior	1/1.3
			Instruction on how to perform the behavior	1/1.3
			Graded tasks	1/1.3

Note: Table 3 presents how inductively derived themes were mapped onto the Process Model of Self-Regulation and corresponding BCTs. Percentages reflect the proportion of references within each theme assigned to each Process Model category or BCT. Although higher-level categories (e.g. Situational Strategies) occur across multiple themes, the specific BCTs and their contextual expressions differ, illustrating how participants applied similar self-regulation processes in distinct ways. In addition, within each theme, Process Model categories are presented in descending order based on the proportion of references assigned to each strategy. Consequently, the order of strategies across themes differs and does not reflect the theoretical sequency of the Process Model.

Participants’ accounts reflected engagement with an average of three of the four Process Model strategies across their interviews. At the lower technique level, a mean of 7.7 BCTs were identified per participant (ranging from 1 to 18). This highlights the wide repertoire of strategies apparent in participants’ accounts of unassisted quitting, in line with the idea that unassisted cessation draws on a wide toolbox of strategies and BCTs.

Together, the inductive themes and deductive analysis reveal the sophisticated ways participants in our study used to self-regulate and succeed in unassisted cessation. Any data not classified under the themes (29/442, 6.6%) are detailed in Appendix 5.

### Theme 1: Doing things differently

Quitting smoking often freed up time in participants’ daily lives, leading them to contemplate how to use their spare time. As Laura expressed, ‘What am I going to do with this time? […] Because it feels like you spend all your time smoking even though you spend a really small percentage of it. I just tried to busy myself.’ This highlights the importance participants placed on finding alternative activities to stay occupied and keep their minds off smoking. Grace echoed this sentiment, stating, ‘I made sure I had something to do. As long as I kept myself busy, I wasn’t too bad.’

Participants engaged in various activities to distract themselves from smoking, including listening to music and chewing gum. Some participants adopted other health behaviors as substitutes for smoking. For example, two participants used vapes to gradually reduce nicotine intake, eventually quitting entirely. Henrietta mentioned, ‘I had my vape in my pocket and only when I was getting really bad cravings, I would get it out and have a puff’, while Grace discussed ‘giving up the habit of smoking, rather than the nicotine’, which she found most challenging. Other common behaviors used to substitute for smoking included snacking, drinking coffee, and engaging in activities like cycling.

Participants also spoke extensively about disrupting their daily routines to quit smoking. For example, Patricia described moving away from the table immediately after eating to avoid the post-meal cigarette. She explained, ‘It’s a disruption of what normally would have gone before, […] we’d [have] enjoyed our dessert, maybe a cup of tea or coffee, and a cigarette. So, if I didn’t do those final steps, then it wouldn’t happen.’ Similarly, Laura noted that she would ‘get up and do the dishes right away’ to disrupt the habit of smoking after dinner. Participants also employed methods to avoid internal triggers, such as watching films instead of regular TV to avoid advert breaks that prompted smoking, removing cigarettes from their pockets, and replacing personal items associated with smoking, such as jumpers and handbags.

Applying the Process Model and BCT taxonomy generated 368 references within Theme 1. Most of the data within this theme (263/368, 71.5%) aligned with the Process Model’s Situational Strategies, including BCTs ‘behavior substitution’ and ‘habit reversal’ (each 128/368, 34.8%), where participants replaced smoking with alternative behaviors. Attention Redeployment accounted for 19.0% (70/368) of the data, with ‘distraction’ commonly cited. Cognitive Change was less frequent (29/368, 7.9%), with BCTs ‘graded tasks’ (18/368, 4.9%) and ‘problem-solving’ (11/368, 3.0%) noted. BCT ‘reducing negative emotions’ was referenced in just 1.6% (6/368) of instances but was not assigned to any Process Model stage. These findings suggest a preference for situational and attentional strategies and BCTs over those targeting cognitive or emotional processes.

### Theme 2: Avoiding tempting situations

Theme 2 reflected participants’ efforts to avoid external situations and social contexts that might tempt them. Participants frequently mentioned the challenge of being around friends who smoked, leading many to avoid specific individuals or social settings. For instance, Henrietta declined to attend a friend’s birthday party, explaining that she was too vulnerable and would likely succumb to smoking if exposed to cigarettes and alcohol. Similarly, Nick avoided friends who smoked, recognizing that continued association would likely result in relapse, stating, ‘I knew if we were still hanging out, I’d fall back into smoking’.

Four participants went as far as relocating. Nick moved to his uncle’s place for seven months, seeking a new environment to re-evaluate his life priorities. He shared, ‘There were so many friends around there […] smoking, and I needed a new environment.’ Ben, on the other hand, avoided places where he used to buy cigarettes and socialize with smokers. He emphasized the importance of changing his ways and taking a break from social interactions to stay focused on his goal. Some participants avoided specific locations linked to smoking. Henrietta stopped shopping at the paper shop where she used to buy cigarettes, explaining, ‘I don’t go near the shop, and it’s just across the street from me. I go somewhere else.’ A few participants avoided staying alone in rooms or enclosed spaces, recognizing isolation as a trigger.

Using the Process Model and BCT taxonomy resulted in 97 references identified under this theme. The Process Model’s Situational Strategies accounted for 94.8% (92/97) of coded data within this theme, with BCT ‘avoiding/reducing exposure to cues’ being most common (68/97, 70.1%). BCTs ‘restructuring the social’ and ‘physical environments’ followed (15/97, 15.5% and 7/97, 7.2% respectively). BCTs ‘behavior substitution’ and ‘habit reversal’ were infrequently used (1/97, 1.0% each). Cognitive Change was rare, with few references to ‘problem-solving’ (5/97, 4.6%).

### Theme 3: Seeking help from friends and family

Participants frequently sought support from friends, family, and colleagues, when facing overwhelming urges to smoke and everyday stresses or anxieties. Most participants expressed gratitude for help and encouragement they received. Several participants reached out to others for support during moments of craving. For example, Grace highlighted the importance of her family and colleagues, stating:

If I was having a bad day, I would speak to whoever happened to be there. I was quite open about the struggle. And it helped me cope with just being able to say that I'm having a really bad day, I really want a cigarette, and they would give me their attention. We would talk, we would do something. It could be as silly as having a giggle at work or something at home, but it took me out of that moment.

Emotional support and encouragement from friends and family was crucial for participants. Nick noted, ‘They would motivate me not to give up, not to lose hope, just to keep pushing, and that I would come out of it a healthy and happy person. That really worked for me, having people during your journey just being very supportive.’ Similarly, Louise shared the influence of her father, stating:

I've always had my dad in my ear, he was half a cheer leader and half sort of coaching me […]. He would tell me about other times where I was having a difficult time at my job in nursing where I was saying ‘I can’t do this’, it was him who pushed me ahead.

Some participants involved friends and family directly in the quitting process by quitting smoking together, thereby providing mutual support. For example, Kirsty quit smoking alongside her friend Nathan, finding it helpful because they both understood the difficulties involved. She noted, ‘I think because me and Nathan had quit at the same time, we had each other’s support. That's what you need when you're doing it—someone who knows how hard it is so you can talk about it.’ Henrietta also quit alongside her daughter, stating, ‘Probably the main person I turned to was my daughter because she gave up the same time as me. […] And she turned to me, and I turned to her. That helped.’

Applying the Process Model and BCT taxonomy generated a total of 73 references within this theme. Situational Strategies from the Process Model accounted for 74.0% (54/73) of the data within this theme, with BCTs ‘social support (emotional)’ most common (39/73, 53.4%), followed by ‘unspecified’ (9/73, 12.3%) and ‘practical’ support (6/73, 8.2%). Cognitive Change comprised 20.5% (15/73) of the references, including ‘verbal persuasion about capability’ (8/73, 11.0%), ‘imaginary reward’ (3/73, 4.1%), and ‘framing/reframing’ (2/73, 2.7%). BCTs ‘commitment’ and ‘imaginary punishment’ were rarely used (1/73, 1.4% each). Attention Redeployment via ‘distraction’ was noted by 3 participants (4/73, 5.5%).

### Theme 4: Gradual ways of quitting smoking

Participants found quitting unassisted challenging due to withdrawal symptoms like headaches, which disrupted their daily activities. As such, they preferred a methodical approach, gradually reducing the number of cigarettes smoked. Aaron captured this sentiment by stating, ‘It’s not easy to just quit in a snap. It’s something you have to do gradually, considering I was not going to see a doctor to give me medication […]. It was a self-quit, so it had to be gradual.’ Similarly, Doug provided a detailed account of his six-month weaning process, illustrating how he gradually decreased his cigarette intake and changed his mindset about smoking. Doug explained:

During the first month, I was used to smoking maybe one packet a day, which is 14 cigarettes. I reduced it to 10 cigarettes a day. The second month, I reduced it to five cigarettes a day. With that gradual change, my body adapted. By the sixth month, I was down to one cigarette a day, and I realized I could go a day without smoking at all. That’s how I ended up quitting completely.

Six participants found financial motivation by gradually saving money previously spent on cigarettes and using it for other purposes, which proved highly rewarding. Patricia transferred her ‘cigarette money’ into a savings account weekly and observed, ‘I could look at that account and see how quickly it was building up. And you know, month one, I thought, wow, my God. And then of course, month two, fantastic! And so on. And then I said, well, this is my next new car.’ Similarly, Mary kept a chart to track the cost of each cigarette and her savings over time. Similarly, some participants emphasized planning and goal setting before gradually reducing cigarette intake. Grace noted, ‘If I’m focused and have a plan, there’s far more chance of carrying it out, as opposed to going ‘right okay, maybe this week I’ll stop smoking’. That just doesn't work for me. But if I've got a plan and I'm focused, it's more likely to succeed.’ Nick echoed, ‘You have to have a workable plan on how you’re going to quit.’

This theme reflected exclusive use of Cognitive Change from the Process Model (67 references total), with notable variation in BCTs. ‘Graded tasks’ were most common (33/67, 49.3%), followed by ‘self-monitoring’ (10/67, 14.9%). Other BCTs included ‘goal setting (behavior)’ (7/67, 10.4%), ‘self-reward’ (5/67, 7.5%), and several less frequent BCTs such as ‘action planning’ and ‘material reward (behavior)’, each cited in 4.5% (3/67) of the data.

### Theme 5: Saying no to cigarettes

This theme captures participants’ experiences declining cigarette offers from friends or colleagues during cessation and examines the implications for their relationships and feelings about their ability to refuse. It also delves into internal struggles when resisting the urge to smoke through thoughts and actions.

Many participants encountered offers of cigarettes during cessation. James, for instance, spoke about the peer pressure from colleagues, mirroring the experience of starting smoking: ‘A lot of our colleagues at work, when we were working together in a team, they all smoked as well. So they were like, here, have one. I was just like, no I don’t want one, I’m not interested. It's kind of like, going way back to when I first started. It's almost like peer pressure when you're at school.’ He described the significance of the first time he declined a cigarette during a night out, noting, ‘When I went home that night I was like, ‘I’ve actually not smoked tonight, I feel quite good about that.’

Declining cigarettes could also be frustrating. Murphy mentioned that his friends treated his decision to quit as a joke, whereas it was a serious commitment for him. He expressed the need to assert his decision: ‘I had to convince them and show them that I'm serious. I don't want to smoke. When we meet, don't give me one, don’t tempt me. You have to respect that.’ For some participants, saying no to cigarettes involved not only verbal refusals but also an internal battle. Kirsty recounted telling herself, ‘don’t smoke’, when tempted. Wayne similarly described his internal thoughts: ‘I kept saying to myself: ‘no cigarettes, I’ve got to do this’. Because of the overall fear. I used to say to myself: ‘is that cigarette really worth lying on my back with them oxygen tanks? No, I don’t want them.’

Applying the Process Model and BCT taxonomy generated a total of 46 references within this theme. Refusing cigarettes was mainly linked to Cognitive Change from the Process Model (38/46, 82.6%), with BCT ‘identity associated with changed behavior’ most frequent (28/46, 60.9%). Less common were ‘framing/reframing’, ‘imaginary punishment’ (each 4/46, 8.7%), and ‘self-talk’ (2/46, 4.3%). Situational Strategies (7/46, 15.2%) included BCTs ‘behavior substitution’, ‘habit reversal’ (each 3/46, 6.5%), and ‘remove access to reward’ (1/46, 2.2%). ‘Distraction’ reflected Attention Re-deployment (1/46, 2.2%).

A small proportion of the dataset (1%) was mapped onto Response Modulation within the Process Model. These instances captured moments where participants directly inhibited their smoking behavior ‘in the moment’. For example, Kirsty’s account of telling herself ‘don’t smoke’ when tempted illustrates this type of immediate self-control. Unlike the Cognitive Change strategies described above, which involved reframing identity or values (‘I’m not a smoker anymore’), or imagining the negative consequences of smoking (‘lying on my back with oxygen tanks’), Response Modulation reflected direct behavioral inhibition without an associated change in one’s identity or perception of smoking. Although infrequent, Response Modulation highlights how participants occasionally drew on momentary restraint to maintain abstinence.

### Theme 6: Setting boundaries, ending friendships, and establishing new ones

Several participants found it necessary to reduce or end relationships with friends whose primary connection was smoking. Emily described this as ‘socially isolating’ herself from certain individuals. Murphy elaborated on the difficulty, stating, ‘I had to stop associating myself with my smoker friends whom we used to smoke together, and it was very hard for me, because we had developed a friendship, a bond. But I had to do that. Because anytime I was with them, I wanted to smoke, and I didn’t want to go back to smoking.’

The decision to end relationships opened opportunities for forming new friendships and integrating into different social groups, reflecting broader changes in participants’ routines and environments. Ben illustrated this shift: ‘I started to mingle with different people in different environments. So rather than going to pubs where there is lots of people from the past to smoke, I started to go to cafes instead. Different social groups, different people, who enjoy coffee, rather than a pint with a cigarette.’ Aaron similarly described making new friends who encouraged healthier activities: ‘I made new friends who would encourage me to do something else apart from [smoking]. I met people who encouraged me to go for hikes together. That drifted my mind away from smoking.’

Four participants mentioned setting boundaries with smoker friends regarding smoking. Henrietta told her friends they could come to her house but had to smoke outside, which her friends respected. Patricia also enforced a no-smoking rule at home, offering a caravan as an alternative smoking area: ‘[…] I say you can go and sit in the caravan and have a cigarette, but I don’t let people smoke in the house. So that helps.’ In addition, Grace adjusted social settings to manage exposure to smoke by ensuring a mix of non-smokers in social gatherings: ‘Even if I was going out with friends for a drink, I made sure that there was a good mix of non-smokers amongst it, so that I wasn’t in a position of being sat at a table by myself or going out to keep them company while they had a cigarette.’

The application of the Process Model and BCT taxonomy yielded 97 references related to this theme. Setting boundaries primarily involved Situational Strategies from the Process Model (34/36, 94.4%), especially BCTs ‘restructuring the social environment’ (23/36, 63.9%) and ‘avoiding/reducing exposure to cues’ (11/36, 30.6%). Cognitive Change was less common (2/36, 5.6%), with ‘goal setting (behavior)’ and ‘commitment’ each cited by one participant (1/36, 2.8%).

### Theme 7: Changing thoughts around smoking

The final theme examines the evolution of participants’ cognitions about smoking during cessation. As Matthew described, ‘[…] smoking is a mind disease; you don’t even have peace of mind.’ Thus, this theme explored how participants altered their thoughts to manage overwhelming urges and withdrawal symptoms, ultimately reshaping their thoughts towards quitting. For example, several participants visualized the adverse consequences of smoking. Amy reflected:

What I was thinking was the feeling in my lungs, […] and that kept me off […] because I knew it would come back. But I did [my] imagination, kind of what I would feel like if I smoked again. I was thinking also of the feeling that I have when I'm walking uphill, and I've just smoked a cigarette where I can’t breathe so freely.

Future-oriented thinking also helped with urges and withdrawal. Matthew, for example, imagined a time when his peace of mind would return: ‘I was imagining one day my peace of mind will be back, and I will be functioning as normal. I will be productive; I'll be able to tackle my problems without struggling, and when I imagined that, I kept on pushing.’ Mary emphasized goal-setting and anticipating obstacles: ‘I believe very much that we can create our own reality. What's the vision that you have in your mind about how this is going to be? What obstacles do you see, what steps are you going to take to overcome the obstacles? And what's the reward at the end of it? It was very goal-directed.’

Self-encouragement and positive self-talk were also significant. Louise gave herself pep talks: ‘having a dialogue where I said, ‘you can do this, Louise, you’ve done it before’. So I tried to be my own cheerleader.’ Finally, Laura adopted a flexible approach to quitting, viewing it as a temporary rather than permanent change. She found this perspective reduced pressure: ‘I thought to myself, if I don’t put any pressure on myself to be a non-smoker, if I have this flexible idea of, I’m stopping now, but I might not be stopped forever, then I’m not putting this massive timeframe where I’m starving myself for this thing that I want.’ This mindset helped her accumulate more smoke-free time and ultimately feel better.

Applying the Process Model and BCT taxonomy generated a total of 77 references within this theme. This theme was only linked to Cognitive Change from the Process Model, with BCT ‘framing/reframing’ being most common (38/77, 49.4%). Other BCTs included ‘imaginary punishment’ (14/77, 18.2%), ‘imaginary reward’ (7/77, 9.1%), ‘salience of consequences’ (5/77, 6.5%), and ‘self-talk’ (4/77, 5.2%). Less frequently reported were ‘problem-solving’, ‘goal setting (outcome)’, ‘information about health consequences’, ‘identity association with changed behavior’, ‘instruction on how to perform the behavior’, and ‘graded tasks’ (each 1 or 2 references out of 77, ≤2.6%).

## Discussion

Our study offers a detailed examination of the diverse self-regulation strategies and behavior change techniques evident in unassisted smoking cessation, moving beyond limiting attributions to the concept of *willpower*. On average, participants’ accounts reflected around seven BCTs, supporting the idea that unassisted cessation involves drawing from a ‘toolbox’ of strategies.

Our qualitative approach uniquely unpacks the concept of willpower in unassisted cessation (Smith et al., 2015a; Stewart, 1999) by focusing on the lived experiences and self-articulated challenges of individuals, allowing us to catalogue the diverse, context-specific strategies they employed. We identified a systematic profile of strategies used to overcome the challenges identified in our inductive analysis. Strategies most often targeted the physical and social environment, aligning with Situation Modification and Situation Selection from the Process Model of Self-Regulation. This finding is consistent with suggestions that smoking is a highly situated habit (Germain et al., 2010; Conklin et al., 2013; Conklin et al., 2008; Stevenson et al., 2017), likely accounting for the popularity of strategies aiming to reduce environmental and interpersonal smoking triggers. Notably, 93.4% of the data was accounted for by the Process Model strategies, and 94.1% by the Behavior Change Taxonomy, suggesting close correspondence between lay participants’ language and expert terminology.

### Implications for our understanding of unassisted cessation

Over half (~45−70%) of smoking cessation is unassisted (Edwards et al., 2014; Smith et al., 2015a; Smoking Toolkit Study, 2024b, 2025), yet understanding *how* individuals quit unassisted has been limited by circular conceptualizations of willpower (Smith et al., 2015a; Stewart, 1999). We overcame this impasse by showing that 99% of the strategies and techniques identified in our study relate to tractable psychological or behavioral processes.

Unassisted quitting often involved altering the situation, accounting for about half of the data (51.9%), and included BCTs such as substituting habitual smoking with other behaviors, avoiding smoking cues, restructuring the social environment, and seeking social support. Cognitive Change strategies, including setting gradual tasks, reframing smoking, adopting a non-smoker identity, and problem-solving, accounted for 28.3% of the data. These findings provide a novel, systematic profile of unassisted cessation strategies through the application of existing self-regulation and behavior change frameworks. Participants reported using strategies spanning three stages of the Process Model of Self-Regulation—Situational Strategies, Attention Redeployment, and Cognitive Change—which could be further specified by the enactment of approximately 7-8 different lower-level BCTs per participant. This breadth of strategies reflects the flexibility and creativity inherent in unassisted cessation, suggesting that individuals draw on diverse techniques to address the multifaceted challenges of quitting.

### Integrating individual- and system-level approaches to self-regulation

In considering self-regulation within the broader context of smoking cessation, it is important to distinguish between individual-level and system-level approaches to understanding quitting, recently identified as i-frames and s-frames, respectively (Chater & Loewenstein, 2023). These frames are sometimes characterized in opposition: i-frames emphasise personal responsibility, while s-frames advocate for societal responsibility.

In relation to unassisted cessation, a self-regulation approach initially appears highly focused on an i-frame. At worst, the tautological definition of willpower is stigmatizing if continued smoking or relapse is viewed as a lack of will. However, unassisted quitters in our study engaged processes that align closely with existing s-framed formal interventions. The Situational Strategies most popular (avoiding triggering situations, replacing smoking with other behaviors) among participants, for example, are a self-deployed analogue of public health measures intended to reduce smoking-cue exposure in the environment, such as packaging and display legislation (Burton et al., 2012; Germain et al., 2010; WHO, 2022). Similarly, Cognitive Change strategies mirror cognitive behavioral therapy and messaging aimed at altering attitudes to smoking. These parallels suggest that i-frame and s-frame approaches may share common mechanisms, rather than suggesting that they operate through potentially antagonistic approaches to understanding public health.

### Integrating self-regulation and behavior change

A secondary goal of our research was to integrate the Process Model of Self-Regulation with the BCT taxonomy. Our results suggest that these approaches are complementary, with the utilisation of both models explaining almost all our data at different levels of abstraction (i.e. from broader strategies to more specific techniques). By integrating BCTs, we introduced a practical, evidence-based approach to classifying these lower-level techniques within an established framework in self-regulation research, providing a practical extension to theoretical work suggesting breaking self-control into these lower-level processes (Werner & Ford, 2023). Our study is the first to apply these BCTs to understand unassisted quitting. We catalogued the 33 BCTs co-occurring with this popular form of quitting, which can be verified and re-used in future observational and experimental work on cessation.

### Limitations, future directions, and potential public health policy implications

While this study offers valuable insights, certain limitations must be acknowledged. The semi-structured guide was informed by the Process Model of Self-Regulation, which may have subtly encouraged participants to discuss their strategies in terms of situational and cognitive processes. However, the design also included open-ended prompts that allowed participants to describe their experiences in their own terms, including direct reflections on response modulation. This deductive-inductive balance combined comprehensive theoretical coverage with maintaining flexibility for participants’ spontaneous narratives. Nonetheless, future research could examine whether alternative framings (e.g. entirely open questions) might elicit different emphases on willpower versus other self-regulatory processes.

It is important to note that, while we have documented the range of strategies people used to quit unassisted, this does not imply these strategies are necessarily the most effective for cessation. For instance, there is ongoing debate regarding the role of e-cigarettes in smoking cessation. Although some participants in our study reported using e-cigarettes as part of their quit attempts, the evidence surrounding their efficacy remains mixed. WHO clinical guidelines on adult tobacco cessation do not currently recommend e-cigarettes as a cessation tool due to insufficient evidence (WHO, 2024). Further, research suggests that e-cigarettes may be more commonly used in place of licensed nicotine products and prescription medication rather than being intrinsically successful in facilitating cessation (Smoking Toolkit Study, 2024a). Thus, more research is needed to clarify the role of e-cigarettes in cessation.

In addition, the present analyses described the processes of self-regulation during unassisted cessation rather than assessing the effectiveness or temporal dynamics of specific strategies. While some participants commented on the helpfulness of different approaches, these reflections were not systematically analyzed, as this was beyond the study’s scope. While the Process Model describes stages of regulatory engagement, our qualitative data were retrospective and not designed to capture temporal sequences of quitting. Consequently, our analysis focused on identifying common challenges and corresponding self-regulation processes rather than the dynamics of change over time. Future research, ideally using longitudinal, diary-based, or experimental designs, could build on these findings by examining how strategies unfold and interact across cessation journeys, and by testing the effectiveness of specific self-regulation strategies.

Key public health policy implications of this study centre on broadening how unassisted cessation is understood and supported within public health frameworks. Our findings suggest that unassisted cessation involves a wide range of self-regulation strategies that can be articulated, modelled, and taught. By identifying concrete, theory-based strategies and BCTs, this study offers a foundation for informing intervention design and communication materials that train these processes. Future policy-oriented work could explore how such strategies might be incorporated into self-help resources, community interventions, or digital support tools. Further research, including intervention development and randomized controlled trials, is needed to translate and implement these strategies effectively.

## Conclusion

Unassisted smoking cessation involves the sophisticated use of multiple self-regulation strategies and BCTs. Unpacking the concept of willpower, participants’ narratives reflected the use of seven-to-eight different techniques on average, indicating that effective self-regulation draws from a diverse ‘toolbox’ of strategies. The most used strategies involved modifying physical and social environments to reduce exposure to smoking triggers, reinforcing the idea that smoking cessation is a deeply situated process. These findings suggest that unassisted quitting is achieved using actionable, teachable strategies, offering a practical framework for understanding and supporting smoking cessation attempts in future interventions.

## Data Availability

Data are available upon request from the corresponding author. Due to the sensitive information around addiction and substance use discussed in the interviews, full transcripts will not be made public. An earlier version of this paper has been posted as a pre-print here.

## Open scholarship

This article has earned the Centre for Open Science badges for Open Materials. The materials are openly accessible at https://osf.io/3r9g2/overview?view_only=0e0992862a564eed810038e00f6330f7; registration of study design and sampling plan of the larger project are openly accessible at https://osf.io/3r9g2/overview?view_only=0e0992862a564eed810038e00f6330f7; registration of the hypotheses and analysis plan are openly accessible at [[--URL--]].
